# How mistimed and unwanted pregnancies affect timing of antenatal care initiation in three districts in Tanzania

**DOI:** 10.1186/1471-2393-13-35

**Published:** 2013-02-06

**Authors:** Amon Exavery, Almamy Malick Kanté, Ahmed Hingora, Godfrey Mbaruku, Senga Pemba, James F Phillips

**Affiliations:** 1 Ifakara Health Institute (IHI), Plot 463, Kiko Avenue, off Old Bagamoyo Road, Mikocheni, P.O. Box 78373, Dar es Salaam, Tanzania; 2 Heilbrunn Department of Population and Family Health, Mailman School of Public Health, Columbia University, New York, USA; 3 Tanzanian Training Centre for International Health (TTCIH), Ifakara, Tanzania

**Keywords:** Mistimed pregnancy, Unwanted pregnancy, Timing, ANC initiation, Tanzania

## Abstract

**Background:**

Early antenatal care (ANC) initiation is a doorway to early detection and management of potential complications associated with pregnancy. Although the literature reports various factors associated with ANC initiation such as parity and age, pregnancy intentions is yet to be recognized as a possible predictor of timing of ANC initiation.

**Methods:**

Data originate from a cross-sectional household survey on health behaviour and service utilization patterns. The survey was conducted in 2011 in Rufiji, Kilombero and Ulanga districts in Tanzania on 3,127 women from whom 910 of reproductive age who had given birth in the past two years and sought ANC at least once during pregnancy were selected for the current analysis. ANC initiation was considered to be early only if it occurred in the first trimester of pregnancy gestation. A recently completed pregnancy was defined as mistimed if a woman wanted it later, and if she did not want it at all the pregnancy was termed as unwanted. Chi-square was used to test for associations and multinomial logistic regression was conducted to examine how mistimed and unwanted pregnancies relate with timing of ANC initiation.

**Results:**

Although 49.3% of the women intended to become pregnant, 50.7% (34.9% mistimed and 15.8% unwanted) became pregnant unintentionally. While ANC initiation in the 1^st^ trimester was 18.5%, so was 71.7% and 9.9% in the 2^nd^ and 3^rd^ trimesters respectively. Multivariate analysis revealed that ANC initiation in the 2^nd^ trimester was 1.68 (95% CI 1.10–2.58) and 2.00 (95% CI 1.05–3.82) times more likely for mistimed and unwanted pregnancies respectively compared to intended pregnancies. These estimates rose to 2.81 (95% CI 1.41–5.59) and 4.10 (95% CI 1.68–10.00) respectively in the 3^rd^ trimester. We controlled for gravidity, age, education, household wealth, marital status, religion, district of residence and travel time to a health facility.

**Conclusion:**

Late ANC initiation is a significant maternal and child health consequence of mistimed and unwanted pregnancies in Tanzania. Women should be empowered to delay or avoid pregnancies whenever they need to do so. Appropriate counseling to women, especially those who happen to conceive unintentionally is needed to minimize the possibility of delaying ANC initiation.

## Background

If sought early, antenatal care (ANC) – health care given to women during pregnancy to ensure healthy outcomes of themselves and their newborns [1] – can be most efficient in averting adverse pregnancy outcomes [2,3]. This is a doorway to early detection and management of potential complications associated with pregnancy, and consequently reduces potential maternal and newborn morbidity and mortality [4-7].

Maternal deaths are caused by complications of abortion, obstetric complications such as haemorrhage, dystocia, eclampsia, sepsis and infections such as tuberculosis and HIV-1, to mention a few [8,9]. Records reveal that haemorrhage and hypertensive disorders account for the largest share of the deaths in developing countries [10]. However, most of these deaths are avertable. For example, proper ANC utilization and skilled attendance at birth have been reported to reduced maternal deaths [4,7]. Also access to emergency obstetric care, adequate nutrition and basic health services considerably lessen the risk of maternal death [11]. Evidence shows moreover that access to essential obstetric care would results into 52% decline in the current global maternal deaths [12].

Unintended pregnancies (mistimed and unwanted) pose important public health risks, and their pernicious consequences have been documented in many studies [13-16]. For example, existing evidence shows presence of a relationship between unintended childbearing and several adverse health outcomes such as maternal depression [17-21], anxiety [20], poor psychological wellbeing [22] and poor utilization of ANC or delivery care [15,16]. Generally, it has been established that women who experience an unintended pregnancy are less likely than women with intended pregnancies to seek care [15,23]. Most of these studies however were conducted in developed countries while such evidence is limited and sometimes inconsistent in developing countries [24].

The World Health Organization (WHO) recommends that adequate care for a normal pregnancy that has no complications should comprise four ANC visits, with the first occurring within the first trimester [25]. The first visit should occur before 12 weeks of gestation but not later than 16 weeks, and afterwards at 24-28 weeks, 32 weeks and 36 weeks [25,26]. Where any complication is detected, frequent visits and if necessary an admission may be advised [2]. Timely execution of the first ANC visit allows more time for more such visits thus guaranteeing timely identification and mitigation of potential pregnancy complications [2] and enables a woman receive a wide range of health promotion and disease curative and preventive services [27]. This includes but not limited to treatment of anemia, diagnosis and treatment of malaria, tuberculosis (TB), sexually transmitted infections (STIs) and tetanus toxoid (TT) immunization. During ANC visits, women also receive counseling on and promotions of skilled attendance at birth, postpartum care for women and newborns and prevention of mother-to-child transmission (PMTCT) of human immunodeficiency virus (HIV) [27].

The situation of ANC in Tanzania is such that, nationwide, the coverage of ANC utilization at least once on the course of pregnancy is near universal. The recent Tanzania Demographic and Health Survey (TDHS) [2] shows that 96% of the Tanzanian women who gave birth five years preceding the survey sought ANC at least once from skilled providers. While this is the case, the first visit (initiation) is often delayed [28] and subsequent visits drop considerably, resulting in incomplete doses of the ANC services such as TT immunization, intermittent preventive treatment of malaria during pregnancy (IPTp) and counseling on birth preparedness. The report shows further that 43% of the women made at least 4 ANC visits recommended by WHO and only 15% made the first ANC visit during the first trimester. The median gestational age at first ANC visit among Tanzanian women was estimated at 5.4 months.

Although women are positive about ANC [28], the health system’s framework for ANC delivery in Tanzania is challenged by factors such as shortages of trained staff, inadequate supply of drugs and equipments [29] and poor implementation of the guidelines of the focused antenatal care (FANC) [30]. While these may affect ANC utilization on one hand, individual factors such as maternal age, maternal education [31] and parity [31,32] are as well acknowledged on the other. Many more studies reporting factors affecting ANC utilization are available [33-38].

Overall, factors affecting ANC utilization are well understood and clearly documented in the literature. The literature classifies ANC utilization in three groups as (1) any ANC (2) ANC initiation and (3) adequate number of ANC visits [15]. Of these groups, the first holds prominent recognition among researchers in both developed and developing countries while the second and third are inadequately researched especially in developing countries. A few studies like a recent one from Kenya on utilization of maternal services among young women (15-24 year-olds) found that place of residence, household wealth, education, ethnicity, parity, marital status and age at birth were associated with both timing of first ANC visit and type of delivery assistance received [3]. One qualitative study from Tanzania reports fear of wild animals on a way to a clinic and lack of money as reasons for late ANC initiation [28]. Another study from Tanzania on timing of ANC initiation found that later ANC enrollment was associated with ethnicity, perceived poor quality of care, late recognition of pregnancy and not being supported by husband or partner [39]. However, a comprehensive review of the predictors of timing of ANC initiation is lacking, since pregnancy intentions – categorized as intended (wanted then), mistimed (wanted later) and unwanted (not wanted at all) – is yet to be recognized as a possible predictor of timing of ANC initiation. Our study therefore responds to this knowledge gap with the following objectives: (1) to quantify the extent of mistimed and unwanted pregnancies, (2) to determine the level of early ANC initiation as a proportion of women initiating ANC in the first trimester, (3) to assess the association between mistimed pregnancy and timing of ANC initiation and (4) to assess the association between unwanted pregnancy and timing of ANC initiation among women of reproductive age who gave birth in the past two years in three districts (i.e. Rufiji, Kilombero and Ulanga) in Tanzania.

## Methods

### Study area

Data for the main survey in which this paper originates were collected in Rufiji, Kilombero and Ulanga districts from May to July 2011. Within these districts, there are two Health and Demographic Surveillance Systems (HDSS) namely; Rufiji HDSS located in Rufiji district, and Ifakara HDSS that occupies portions of Kilombero and Ulanga districts. The survey was based on the population under surveillance by these HDSS of which at the time of the survey, they were altogether made up of 101villages (38 in Rufiji, and 63 in Ifakara) with over 350,000 people. These HDSS are broadly described elsewhere [40-43].

### Study design and sampling

The parent study was designed as a cross-sectional survey of random households. Further details of the survey are available elsewhere [44].

### Data collection

Data collection was conducted by the Ifakara Health Institute (IHI) in Tanzania in collaboration with the Mailman School of Public Health of Columbia University (MSPH/CU), USA, for *Connect*, a 5-year project which is being implemented in Kilombero, Rufiji and Ulanga districts in Tanzania. The project is further described at http://www.ihi.or.tz/projects/the-connect-project. Data collection procedures, questionnaire modules and the purpose of the survey are described elsewhere [44].

### Study population

Women of reproductive age (15-49 years) who had given birth in the past two years preceding the survey and had sought ANC at least once during pregnancy were eligible for the current analysis, thus extracted from the parent database. Seeking ANC at least once was considered a relevant criterion because more than 99% (n = 910) of all the women who had given birth in the past two years actually did so. Women who reported having never sought any ANC during pregnancy were exceptionally few (<1%) thus excluded from the analysis.

### Study variables

#### Dependent variable

Our dependent variable was ‘Timing of ANC initiation’. As already pointed out [1], ANC initiation in the first trimester is very crucial. This leads to complete doses of various immunizations such as TT and enables timely detection and management of possible pregnancy complications, thus resulting in desirable health outcomes for women and newborns. Therefore, all first ANC visits that occurred within the first trimester (1 trimester = 12 weeks [25]) were operationally referred to as ‘early ANC initiation’ and those that occurred afterwards – during the 2^nd^ and 3^rd^ trimester – were referred to as ‘late ANC initiation’. This definition has been similarly applied elsewhere [3]. This variable thus had three categories as 1^st^ trimester, 2^nd^ trimester and 3^rd^ trimester which were statistically represented by codes ‘1’, ‘2’ and ‘3’ respectively for computational purposes.

#### Independent variables

The main independent variable was pregnancy intentions for a recently completed pregnancy (birth within 2 years) and had three categories as (1) intended (pregnancy wanted at the time of conception), (2) mistimed (pregnancy wanted later) and (3) unwanted (pregnancy not wanted at all). Other independent variables included were age (<20, 20-34 and >34), marital status (married, ever married and single), education (none, primary/higher), religion (Christian, Muslim and other) and ethnicity (Ndengereko, Ngindo, Pogoro, Sukuma and Others). Household wealth with three categories as poor, middle and rich was created using principal component analysis (PCA) [45] based on household asset ownership of a toilet, type of the toilet and source of drinking water. We also included travel time (in hours) to a health facility (<0.5, 0.5-1 and >1) which was a self-reported variable requiring a woman to report how much time it took her to get to a place where she received last ANC during pregnancy. We further included district of residence (Kilombero, Rufiji, and Ulanga), type of residence (rural and urban) and gravidity (1, 2-4 and >4). Gravidity was considered as a proxy for fertility.

### Statistical analysis

Simple frequency distribution of the sample for each of the variables involved was conducted. Timing of ANC initiation was then cross-tabulated against pregnancy intentions, and the rest of the independent variables. Since all variables were categorical, the degree of association between each pair was tested using Chi-Square.

Finally, a multinomial (polytomous) logistic regression was conducted to examine how pregnancy intentions affected timing of ANC initiation, controlling for potential confounding variables. The model was chosen because the dependent variable had multiple categories which were assumed to have no natural ordering, and even if they did as Magadi et al [3] shows, the information conveyed by the ordered nature had no meaningful use given the objectives. The baseline or reference category was 1^st^ trimester, thus elucidating how likely it was a pregnant woman to initiate ANC in the 2^nd^ and 3^rd^ trimester relative to the 1^st^ trimester. In this process, age, gravidity and travel time were treated as continuous to ensure efficiency of the model [46]. Selection of the variables for the multivariate model was made using log-likelihood ratio test [47], except the main independent variable of interest. This meant that in order to end-up with a parsimonious model, a variable was retained in the multivariate model only if there was adequate statistical justification that its presence improved the overall model. The entire process of data analysis was handled in STATA (version11) statistical software.

### Ethical consideration

An ethical approval to conduct the main survey was given by the Medical Research Coordinating Committee (MRCC) of the National Institute for Medical Research (NIMR) in Tanzania. The Ifakara Health Institute (IHI)’s Institutional Review Board (IRB) also approved the study. Participation in the survey was perfectly voluntary. All women aged 18 and above signed an informed consent first after which they were interviewed. Similarly, an assent was sought from those that were under 18 years of age. Women below 15 years of age were not included. Storage of the completed questionnaires was separate from the consent and assent forms to ensure anonymity of the data. The whole process of data collection and handling was confidential and managed by a few experts.

## Results

### Sample characteristics

As Table 1 shows, the participants were 910 women ages 15-49 years (mean = 27.9, SD = 7.6) who had given birth in the past two years and had received ANC at least once during pregnancy. Majority of the women were married or in consensual unions (79.9%), 5.5% ever married (widowed or divorced) and 14.6% were single. About a quarter (24.3%) of the women had no formal education and the rest (75.7%) had primary or higher education. With regard to religion, Islam and Christianity constituted a majority of the participants (51.2% and 42.7% respectively). Concerning ethnic groups, more than 50 tribes were recorded, although many of them were very small thus combining such into a single category as ‘Others’. These were 15.2% Ndengereko, 14.3% Ngindo, 14.0% Pogoro, 11.0% Sukuma and 45.5% Others. In terms of household wealth, women were distributed in the three household wealth categories as 40.5% poor, 35.1% middle and 24.5% rich. More than a half (58.7%) of all participants resided in Kilombero district.

**Table 1:** Sample characterstics (N = 910)

**Variable**	**n**	**%**
**Timing of ANC initiation**		
1^st^ trimestert (early)	168	18.5
2^nd^ trimestert (late)	652	71.7
3^rd^ trimestert (very late)	90	9.9
Median = 5.0 months	-	-
**Pregnancy intentions**^**m**^		
Intended	429	49.3
Mistimed	304	34.9
Unwanted	137	15.8
**Gravidity**		
1	184	20.2
2-4	430	47.3
>4	296	32.5
Median = 3, IQR = 3	-	-
**Age (years)**		
<20	134	14.7
20-34	557	61.2
>34	219	24.1
Mean = 27.9, SD = 7.6	-	-
**Marital status**^**m**^		
Married	726	79.9
Ever married	50	5.5
Single	133	14.6
**Education**		
None (never been to school)	221	24.3
Primary/higher	689	75.7
**Religion**^**m**^		
Christian	388	42.7
Muslim	465	51.2
Other (e.g. traditional)	55	6.1
**Ethnicity**		
Ndengereko	138	15.2
Ngindo	130	14.3
Pogoro	127	14.0
Sukuma	101	11.0
Others	414	45.5
**Household wealth**^**m**^		
Poor	337	40.5
Middle	292	35.1
Rich	204	24.5
**Travel time to health facility (hours)**^**m**^		
<0.5	195	21.9
0.5-1	260	29.2
>1	436	48.9
Mean = 1.05, SD = 0.92	-	-
**District of residence**		
Kilombero	534	58.7
Rufiji	224	24.6
Ulanga	152	16.7
**Type of residence**^**m**^		
Rural	737	81.2
Urban	171	18.8
**Total**	910	100.0

^m^Variable categories do not add-up to 910 due to missing.

### Timing of ANC initiation

**Figure 1:**
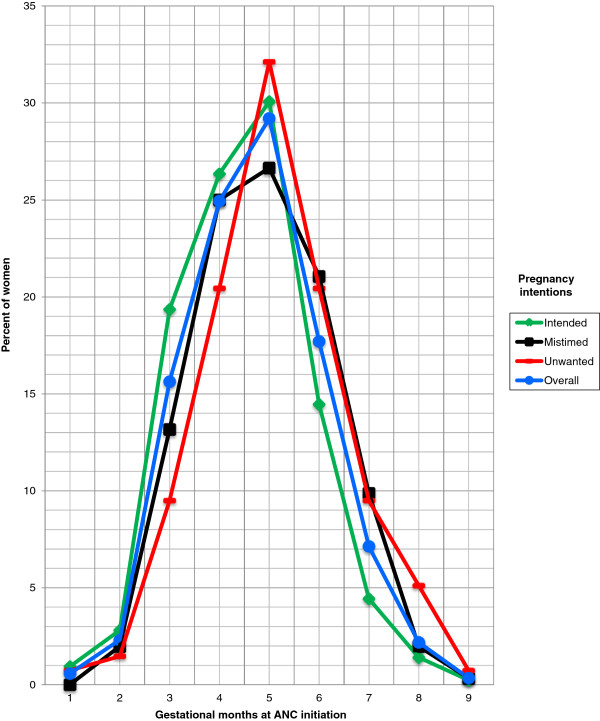
Timing of ANC initiation by pregnancy intentions among 15-49 year-old women who had given birth in the past two years in three districts in Tanzania, 2011 (n = 870).

Slightly less than 1 in every 5 women (18.5%) made the first ANC visit in the first trimester. The rest, 81.5% (71.7% in the 2^nd^ trimester, and 9.9% in the 3^rd^ trimester), delayed ANC initiation. ANC initiation in the first four months stood at 43.4% (Figure 1). Median gestational age at first ANC visit was 5.0 months. Regarding pregnancy intentions, 49.3% of all the women reported that they intended to become pregnant at the time of conception; whereas 34.9% mistimed their pregnancies and 15.8% did not want to become pregnant at all. Therefore 50.7% of all women who gave birth in the past two years in the study area became pregnant unintentionally.

Figure 1 shows timing of ANC initiation by pregnancy intentions. Early ANC initiation was highest for intended pregnancies but lowest for unwanted and mistimed pregnancies. Of the curves showing the proportion of women initiating ANC, the highest was for intended pregnancies while the lowest was for unwanted pregnancies in the first four months of pregnancy, but the difference was subtle in first two months. The curve representing mistimed pregnancies was the second lowest in the same period. ANC initiation pinnacled in the 5^th^ month of gestation for all levels of pregnancy intentions, but was highest for unwanted pregnancies and lowest for mistimed pregnancies. The situation observed in the first four months was reversed in the last four months, with women who had intended pregnancies being the fewest for ANC initiation while those who had unwanted pregnancies led. Timing of ANC initiation for mistimed pregnancies followed the same pattern as for unwanted pregnancies and the two become the same during the 6-7^th^ month of gestation.

**Figure 2:**
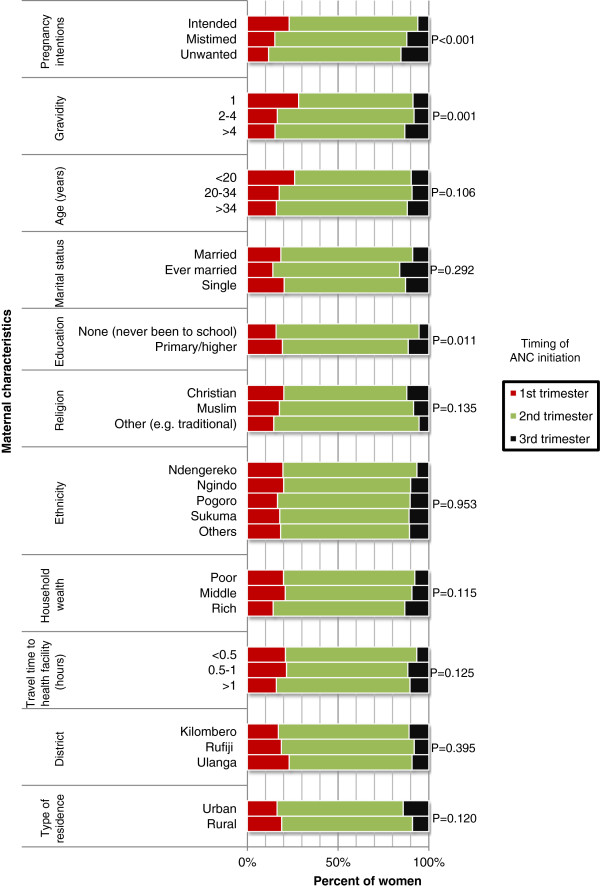
Timing of ANC initiation by maternal characteristics among 15-49 year-old women who had given birth in the past two years in three districts in Tanzania, 2011 (n = 910).

Figure 2 presents bivariate analysis of timing of ANC initiation by maternal characteristics. The results show that timing of ANC initiation was significantly associated with pregnancy intentions (P<0.001), such that ANC initiation in the 1^st^ trimester was highest (23.1%) among women who became pregnant intentionally, but dropped considerably to 15.1% and 11.7% among women whose pregnancies were mistimed and unwanted respectively. The results show further that the higher the gravidity the lower was the proportion of women initiating ANC early (P = 0.001). More than a quarter (28.3%) of gravida one women initiated ANC in the 1^st^ trimester. This proportion was 16.5% and 15.2% among women whose gravidity was 2-4 and >4 pregnancies respectively. Education was also associated with ANC initiation (P = 0.011) with early ANC initiation being 19.3% among women who had primary education or higher and 15.8% among those who had no formal education. ANC initiation by other characteristics is presented in Figure 2 with no significant differences (P>0.05) across various categories.

### Multinomial logistic regression results

Further analysis was conducted in a multivariable fashion using multinomial logistic regression to examine how mistimed and unwanted pregnancies relate with ANC initiation, controlling for possible confounders. Relative risk ratios (RRR) and their corresponding 95% confidence intervals were calculated and presented in Table 2 in which the effect of pregnancy intentions as a predictor of interest on timing of ANC initiation is adjusted for gravidity, age, education, household wealth, marital status, religion, district of residence and travel time to a health facility.

**Table 2:** **Multivariate polytomous logistic regression of factors associated with ANC initiation in the 2**^**nd **^**and 3**^**rd **^**trimesters relative to 1**^**st **^**trimester in three districts in Tanzania, 2011 (n = 777)**

	**ANC initiation in the 2**^**nd **^**trimester relative to 1**^**st **^**trimester**		**ANC initiation in the 3 **^**rd**^**trimester relative to 1**^**st **^**trimester**	
**Variable**	**Related risk ratio (RRR)**	**95% confidence interval (CI)**	**Related risk ratio (RRR)**	**95% confidence interval (CI)**
**Pregnancy intensions**				
Intended^a^	1.00	-	1.00	-
Mistimed	1.68**	1.10-2.58	2.81	1.41-5.59
Unwanted	2.00*	1.05-3.82	4.10***	1.68-10.00
**Gravidity-Age intentions**				
Gravidity	1.58*	1.04-2.39	2.27**	1.21-4.27
Age	1.06*	1.01-1.12	1.04	0.96-1.13
Gravidity*Age	0.99	0.98-1.00	0.98*	0.97-1.00
**Education**				
None (never been to school)^a^	1.00	-	1.00	-
Primary/higher	0.83	0.50-1.38	2.12	0.81-5.57
**Household wealth**				
Poor^a^	1.00	-	1.00	-
Middle	1.11	0.71-1.75	1.31	0.62-2.77
Rich	1.73	0.98-3.06	2.38*	1.01-5.64
**Marital status**				
Married^a^	1.00	-	1.00	-
Ever married	1.45	0.53-3.95	3.09	0.86-11.08
Single	1.21	0.69-2.11	1.61	0.15-17.79
**Religion**				
Christian^a^	1.00	-	1.00	-
Muslim	1.31	0.85-2.02	0.9	0.46-1.79
Other (e.g. traditional)	2.41	0.71-8.21	1.61	0.15-17.79
**District**				
Kilombero^a^	1.00	-	1.00	-
Rufiji	0.97	0.55-1.69	0.90	0.36-2.26
Ulanga	0.66	0.38-1.12	0.45	0.17-1.16
**Travel time to health facility (hours)**	1.23	0.95-1.59	1.26	0.86-1.84

***P<0.01, **P<0.02, *P<0.05; ^a^Reference/baseline category.

The findings reveal that ANC initiation in the second trimester was significantly and independently 1.68 times more likely for mistimed pregnancies compared to intended pregnancies (RRR = 1.68, 95% CI 1.10–2.58). Similarly, ANC initiation in the second trimester was 2.00 times more likely for unwanted pregnancies compared to intended pregnancies (RRR = 2.00, 95% CI 1.05–3.82). On the other hand, ANC initiation in the third trimester resembled the pattern observed in the second trimester, such that women who had mistimed and unwanted pregnancies were 2.81 (95% CI 1.41–5.59) and 4.10 (95% CI 1.68–10.00) respectively times more likely to initiate ANC in the third trimester compared to women whose pregnancies were intended.

There was an age–gravidity interaction in the prediction of ANC initiation in the second and third trimester. At younger ages, the relative risk of ANC initiation in the second trimester was 1.58 times higher for every one pregnancy increase in gravidity (RRR = 1.58, 95% CI 1.04–2.39). This risk was 2.27 times higher in the third trimester (RRR = 2.27, 95% CI 1.21–4.27). At lower values of gravidity, a one-year increase in age was significantly associated with a 6% increase in the relative risk of ANC initiation in the second trimester (RRR = 1.06, 95% CI 1.01–1.12). The risk was 4% in the third trimester but was not statistically significant (RRR = 1.04, 95% CI 0.96–1.13). In contrast, a one pregnancy increase in gravidity was significantly associated with 1% less risk of ANC initiation in the second trimester for older women than it was for younger women (RRR = 0.99, 95% CI 0.98–1.00). Similarly, the risk of ANC initiation in the third trimester was 2% lower for every one pregnancy increase in gravidity at older ages than it was at younger ages (RRR = 0.98, 95% CI 0.97–1.00).

Women from socioeconomically rich households were significantly 2.38 times more likely to initiate ANC in the third trimester compared to women from poor households (RRR = 2.38, 95% CI 1.01–5.64).

## Discussion

This study shows that late ANC initiation in Kilombero, Rufiji and Ulanga districts in Tanzania is extensive. Late ANC initiation of 81.5% observed is very high but slightly lower than the national estimate of 85.0% [2]. It is also lower than 89.0% observed in Kenya [3]. The median gestation of 5.0 months at first ANC visit observed was also comparable to the national estimate of 5.4 [2], indicating that a half of the women sought their first ANC after the 5^th^ month of pregnancy gestation. Delaying ANC initiation threatens maternal and newborn health for it deprives women’s full benefits of effective ANC [48]. It forfeits the opportunity for timely diagnosis and identification of possible pregnancy-related problems thus delaying care for enhancing women and newborn health [1]. Deliberate efforts are therefore needed to uplift early ANC initiation. This is important and may be a prerequisite to attaining the adequate number of ANC visits that WHO recommends for enhanced health outcomes of mothers and newborns [25].

Regarding pregnancy intentions, almost a half of all the women who had given birth in the past two years in the study area became pregnant unintentionally. This figure was nearly twice as high as the national estimate [2]. Our regressions confirmed a significant delay in ANC initiation for both mistimed and unwanted pregnancies compared to intended pregnancies. The risk of delaying ANC initiation was even much higher and stronger in the third trimester. Also, the risk of delaying ANC initiation was higher for unwanted than that for mistimed pregnancies in both second and third trimesters. Women who experience mistimed or unintended pregnancies may be missing the support (such as familial or partner support) for good health care seeking behaviour. Therefore improving broad community support for health care seeking during pregnancy may benefit these women. Some studies have found that women with unintended pregnancies do not seek ANC as early as do those with planned pregnancies because of late recognition of pregnancy [39] or delay in deciding whether or not to terminate the pregnancy [13]. Although there exists no sufficient evidence of any workable intervention that can increase early ANC initiation [49], alternatives should be devised to rescue the situation. While it is important to adequately counsel women concerning positive mind-sets about ANC for both intended and unintended pregnancies, proper utilization of family planning services should be encouraged in order to reduce the current level of unmet need in Tanzania which is presently one of the highest in the world [2]. If this is successful, a massive reduction in the current level of unintended pregnancies may be expected. Preventing unintended pregnancies is very crucial for enhanced wellbeing of women and their newborns on one hand and may beneficially affect population size on the other [50,51].

These findings imply that mistimed and unwanted pregnancies represent an elevated risk of adverse health outcomes as far as maternal and newborn health is concerned. Considering also that induced abortion is illegal in Tanzania, mistimed and unwanted pregnancies may lead to unsafe abortions as women will most likely not obtain proper care. The level of maternal mortality in Tanzania may thus be under estimates since deaths arising from unsafe abortions may be underreported. Women’s behavioural and attitudinal outlook of ANC utilization should be changed especially when they conceive unintentionally.

Although young multi–gravid women and older women with fewer pregnancies delayed ANC initiation as similarly observed elsewhere [16,52], older multi–gravid women were unlikely to do so. The interaction between age and gravidity has also been found to exist in the prediction of other health outcomes apart from timing of ANC initiation [53]. Less risk of delaying ANC initiation observed among older multi–gravid women is possibly due to positively shaped mindsets of ANC from health education sessions that women may have had in previous pregnancies, as well as perceived risk of pregnancy complications at age 35 or older [54]. However, underlying mechanisms of this observation remain less clear. Similar studies did not include an interaction term between age and gravidity or parity, and they report that multi-gravid women delay ANC initiation due to confidence they tend to develop from experience accrued from past pregnancies and they sometimes believe that modern health care makes little or no change [31]. There is also a belief that pregnancy complications and their associated difficulties occur mostly among young women [31].

It was unexpected as we found in this study that women from socioeconomically rich households would delay ANC initiation. This observation differs from findings of one qualitative study conducted in Tanzania that found among other things that lack of money delays ANC initiation [28]. It is however possible that rich women may delay in deciding where to seek ANC (selectivity) due to perceived quality of care thus end-up delaying ANC initiation. Considering these inconsistencies, a more focused investigation may be required to reveal how socioeconomic status actually relates with timing of ANC initiation in Tanzania and beyond.

### Limitations

We admit absence of some important variables such as family size and pregnancy recognition which would alight more on the question at hand. There were some missing data for some variables, although this in general did not affect results and interpretations. No causal inferences may be drawn from these findings due to obvious difficulties in ascertaining temporality in cross-sectional surveys. Also there may have been a recall bias effect because the study relied on women’s retrospective recall of the timing of their ANC initiation. Since three districts only were studied, findings may not be generalized countrywide and beyond. A qualitative study may be needed to explore both individual and social perceptions, beliefs, norms and other barriers against early ANC initiation especially with mistimed and unwanted pregnancies in Tanzania.

## Conclusion

A significant delay in ANC initiation is confirmed as an adverse maternal and child health consequence of mistimed and unwanted pregnancies in Tanzania. Women should be empowered to be able to delay or avoid pregnancies whenever they need to do so. Use of family planning services should be emphasized in order to reduce and eventually eradicate unintended pregnancies. In addition, efforts should be made to change women’s health seeking behaviour concerning ANC utilization especially when they conceive unintentionally. Couples and the community at large should know that improper ANC utilization endangers not only the pregnancy but also the woman’s own life.

On the part of the *Connect* Project, training of the Community Health Agents (CHA) should emphasize further on establishing from a pregnant woman whether her pregnancy was intended or unintended. This would assist in providing appropriate counseling to the woman and thus minimize the possibility of delaying ANC initiation. The CHAs are better positioned to intervene timely as they visit these women early and regularly in their households.

## Competing interest

The authors declare that they have no competing interests.

## Authors’ contributions

AE conceptualized the problem, designed the study, performed data analysis and wrote the manuscript drafts. AMK participated in designing the study, data analysis and critical review of the manuscript. AH, GM and SP critically reviewed the manuscript. JFP participated in designing the study and critical review of the manuscript. All authors read and approved the final draft.

## Pre-publication history

The pre-publication history for this paper can be accessed here:

http://www.biomedcentral.com/1471-2393/13/35/prepub

## References

[B1] World Health Organization (WHO) United Nations Children's Fund (UNICEF) Antenatal care in developing countries: promises, achievements and missed opportunities - an analysis of trends, levels and differentials, 1990-2001 2003 Geneva: WHO

[B2] National Bureau of Statistics (NBS) [Tanzania], ICF Macro Tanzania demographic and health survey 2010 2011 Dar es Salaam, Tanzania: NBS and ICF Macro

[B3] Ochako R Fotso J Ikamari L Khasakhala A Utilization of maternal health services among young women in Kenya: insights from the Kenya demographic and health survey, 2003 BMC Pregnancy Childbirth 2011 11 1 10.1186/1471-2393-11-1 21214960 PMC3022772

[B4] Magadi M Madise N Diamond I Factors associated with unfavourable birth outcomes in Kenya J Biosoc Sci 2001 33 199 225 10.1017/S0021932001001997 11284627

[B5] Mpembeni RMN Killewo JZ Leshabari MT Siriel N Massawe SN Jahn A Mushi D Mwakipa H Use pattern of maternal health services and determinants of skilled care during delivery in Southern Tanzania: implications for achievement of MDG-5 targets BMC Pregnancy Childbirth 2007 7 29 10.1186/1471-2393-7-29 18053268 PMC2222241

[B6] Reynolds HW Wong EL Tucker H Adolescents' use of maternal and child health services in developing countries Int Fam Plan Perspect 2006 32 6 16 10.1363/3200606 16723297

[B7] UNICEF Eastern and Southern Africa Regional Office: Maternal Mortality Reduction Strategy 2003 http://www.popline.org/node/526231. Feb 5, 2013

[B8] Inter-Agency Group for Safe Motherhood The safe motherhood action agenda: priorities for the next decade. Report on the safe motherhood technical consultation 18-23 October 1997 1997 Colombo, Sri Lanka http://www.popline.org/node/526231. Feb 5, 2013

[B9] Khan M Pillay T Moodley JM Connolly CA Maternal mortality associated with tuberculosis-HIV-1 co-infection in Durban South Africa. AIDS 1863 2001 15 10.1097/00002030-200109280-00016 11579249

[B10] Khan KS Wojdyla D Say L Gülmezoglu AM Van Look PF WHO analysis of causes of maternal death: a systematic review Lancet 2006 367 1066 1074 10.1016/S0140-6736(06)68397-9 16581405

[B11] UNICEF Progress for children: a report card on maternal mortality http://www.unicef.org/childsurvival/files/Progress_for_Children-No._7_Lo-Res_082008.pdf. 2008. 7-23-2012

[B12] Wagstaff A Claeson M The millennium development goals for health rising to the challenges 2004 Washington DC: The World Bank

[B13] Kost K Landry DJ Darroch JE Predicting maternal behaviors during pregnancy: does intention status matter? Fam Plann Perspect 1998 30 79 88 10.2307/2991664 9561873

[B14] Gage AJ Premarital childbearing, unwanted fertility and maternity care in Kenya and Namibia Popul Stud 1998 52 21 34 10.1080/0032472031000150156

[B15] Eggleston E Unintended pregnancy and women's use of prenatal care in Ecuador Soc Sci Med 2000 51 1011 1018 10.1016/S0277-9536(00)00010-1 11005389

[B16] Magadi MA Madise NJ Rodrigues RN Frequency and timing of antenatal care in Kenya: explaining the variations between women of different communities Soc Sci Med 2000 51 551 561 10.1016/S0277-9536(99)00495-5 10868670

[B17] Lau Y Keung DW Correlates of depressive symptomatology during the second trimester of pregnancy among Hong Kong Chinese Soc Sci Med 2007 64 1802 1811 10.1016/j.socscimed.2007.01.001 17316942

[B18] Nakku JN Nakasi G Mirembe F Postpartum major depression at six weeks in primary health care: prevalence and associated factors Afr Health Sci 2006 6 207 214 17604509 10.5555/afhs.2006.6.4.207 PMC1832062

[B19] Barber JS Axinn WG Thornton A Unwanted childbearing, health, and mother-child relationships J Health Soc Behav 1999 40 231 257 10.2307/2676350 10513146

[B20] Najman JM Morrison J Williams G Andersen M Keeping JD The mental health of women 6 months after they give birth to an unwanted baby: a longitudinal study Soc Sci Med 1991 32 241 247 10.1016/0277-9536(91)90100-Q 2024133

[B21] Lara MA Navarro C Navarrete L Cabrera A Almanza J Morales F Juárez F Depressive symptoms in pregnancy and associated factors in patients of three health institutions in Mexico City Salud Mental 2006 29 55 62

[B22] Hardee K Eggleston E Wong EL Irwanto Hull TH Unintended pregnancy and women's psychological well-being in Indonesia J Biosoc Sci 2004 36 617 626 10.1017/S0021932003006321 15446355

[B23] Marston C Cleland J Do unintended pregnancies carried to term lead to adverse outcomes for mother and child? an assessment in five developing countries Popul Stud 2003 57 77 93 10.1080/0032472032000061749 12745811

[B24] Gipson JD Koenig MA Hindin MJ The effects of unintended pregnancy on infant, child, and parental health: a review of the literature Stud Fam Plann 2012 39 18 38 18540521 10.1111/j.1728-4465.2008.00148.x

[B25] Villar J Ba'aqeel H Piaggio G Lumbiganon P Miguel Belizán J Farnot U Al-Mazrou Y Carroli G Pinol A Donner A Langer A Nigenda G Mugford M Fox-Rushby J Hutton G Bergsjø P Bakketeig L Berendes H Garcia J WHO Antenatal Care Trial Research Group WHO antenatal care randomised trial for the evaluation of new model of routine antenatal care Lancet 2001 357 1551 1564 10.1016/S0140-6736(00)04722-X 11377642

[B26] Villar J Bergsjo P WHO antenatal care randomized trial: manual for the implementation of the New model. WHO/RHR/01.30 2003 WHO: Geneva

[B27] Maternal and Child Health Division Focused antenatal care: providing integrated, individualized care during pregnancy http://www.accesstohealth.org/toolres/pdfs/ACCESStechbrief_FANC.pdf. 2007. 7-22-2012

[B28] Mrisho M Obrist B Schellenberg JA Haws RA Mushi AK Mshinda H Tanner M Schellenberg D The use of antenatal and postnatal care: perspectives and experiences of women and health care providers in rural southern Tanzania BMC Pregnancy Childbirth 2009 9 10 10.1186/1471-2393-9-10 19261181 PMC2664785

[B29] Nyamtema AS Jong AB Urassa DP Hagen JP van Roosmalen J The quality of antenatal care in rural Tanzania: what is behind the number of visits? BMC Pregnancy Childbirth 2012 12 70 10.1186/1471-2393-12-70 22823930 PMC3434089

[B30] Gross K Schellenberg JA Kessy F Pfeiffer C Obrist B Antenatal care in practice: an exploratory study in antenatal care clinics in the Kilombero Valley, south-eastern Tanzania BMC Pregnancy Childbirth 2011 11 36 10.1186/1471-2393-11-36 21599900 PMC3123249

[B31] Regassa N Antenatal and postnatal care service utilization in southern Ethiopia: a population-based study Afr Health Sci 2011 11 390 397 22275929 PMC3260999

[B32] Agus Y Horiuchi S Factors influencing the use of antenatal care in rural West Sumatra, Indonesia BMC Pregnancy Childbirth 2012 12 9 10.1186/1471-2393-12-9 22353252 PMC3298506

[B33] Iyaniwura CA Yussuf Q Utilization of antenatal care and delivery services in Sagamu, south western Nigeria Afr J Reprod Health 2009 13 111 122 20690266

[B34] Tura G Antenatal care service utilization and associated factors in Metekel zone, northwest Ethiopia Ethiop J Health Sci 2009 19 2

[B35] Ye Y Yoshida Y Harun-Or-Rashid MD Sakamoto J Sakamoto J Factors affecting the utilization of antenatal care services among women in Kham District, Xiengkhouang province Lao PDR Nagoya J Med Sci 2010 72 33 PMC11254371 20229700

[B36] Simkhada B Teijlingen ER Porter M Simkhada P Factors affecting the utilization of antenatal care in developing countries: systematic review of the literature J Adv Nurs 2008 61 244 260 10.1111/j.1365-2648.2007.04532.x 18197860

[B37] Tewodros B Mariam AG Dibaba Y Factors affecting antenatal care utilization in Yem special woreda southwestern Ethiopia Ethiop J Health Sci 2009 19 1

[B38] Dairo MD Owoyokun KE Factors affecting the utilization of antenatal care services in Ibadan, Nigeria Benin J Postgrad Med 2010 12 1

[B39] Gross K Alba S Glass TR Schellenberg JA Obrist B Timing of antenatal care for adolescent and adult pregnant women in south-eastern Tanzania BMC Pregnancy Childbirth 2012 12 16 10.1186/1471-2393-12-16 22436344 PMC3384460

[B40] Shabani J Lutambi AM Mwakalinga V Masanja H Clustering of under-five mortality in Rufiji Health and Demographic Surveillance System in rural Tanzania Glob Health Action Supplement 1 2010 3 http://www.ncbi.nlm.nih.gov/pmc/articles/PMC2935925/pdf/GHA-3-5264.pdf 10.3402/gha.v3i0.5264 PMC2935925 20838634

[B41] Schellenberg JR Abdulla S Minja H Nathan R Mukasa O Marchant T Mponda H Kikumbih N Lyimo E Manchester T Tanner M Lengeler C KINET: a social marketing programme of treated nets and net treatment for malaria control in Tanzania, with evaluation of child health and longterm survival Trans R Soc Trop Med Hyg 1999 93 225 231 10.1016/S0035-9203(99)90001-9 10492745

[B42] Hetzel MW Iteba N Makemba A Mshana C Lengeler C Obrist B Schulze A Nathan R Dillip A Alba S Mayumana I Khatib RA Njau JD Mshinda H Understanding and improving access to prompt and effective malaria treatment and care in rural Tanzania: the ACCESS Programme Malar J 2007 6 83 10.1186/1475-2875-6-83 17603898 PMC1925101

[B43] Gross K Alba S Schellenberg J Kessy F Mayumana I Obrist B The combined effect of determinants on coverage of intermittent preventive treatment of malaria during pregnancy in the Kilombero Valley Tanzania Malar J 2011 10 140 10.1186/1475-2875-10-140 PMC3126755 21599999

[B44] Exavery A Kanté AM Jackson E Noronha J Sikustahili G Tani K Mushi HP Baynes C Ramsey K Hingora A Phillips JF Role of condom negotiation on condom use among women of reproductive age in three districts in Tanzania BMC Publ Health 2012 12 1097 10.1186/1471-2458-12-1097 PMC3585459 23256530

[B45] Vyas S Kumaranayake L Constructing socio-economic status indices: how to use principal components analysis 2006; 21 (6): 459-468 Health Policy Plan 2006 21 459 468 10.1093/heapol/czl029 17030551

[B46] de Leon AR Soo A Williamson T Classification with discrete and continuous variables via general mixed-data models J Appl Stat 2011 38 1021 1032 10.1080/02664761003758976

[B47] Vittinghoff E Shiboski SC Glidden DV McCulloch CE Dietz K, Gail M, Krickeberg K, Samet J, Tsiatis A Predictor selection Regression methods in biostatistics: linear, logistic, survival, and repeated measures models 2005 New York, USA: Springer Science+Business Media, Inc 133 156

[B48] Oluwatosin OA Aluko JO Onibokun A Factors influencing initiation of antenatal care in Ibadan, Nigeria Afr J Midwifery Womens Health 2011 5 163 168

[B49] Oakley L Gray R Kurinczuk JJ Brocklehurst P Hollowell J P14 Interventions to increase the early initiation of antenatal care in socially disadvantaged and vulnerable women: a systematic review J Epidemiol Community Health 2010 64 A39

[B50] Yohannes S Wondafrash M Abera M Girma E Duration and determinants of birth interval among women of child bearing age in Southern Ethiopia BMC Pregnancy Childbirth 2011 11 38 10.1186/1471-2393-11-38 21599927 PMC3112064

[B51] Bongaarts J The proximate determinants of unwanted childbearing in the developing world 1997 America: Paper presented at the Annual Meeting of Population Association of

[B52] Fotso JC Ezeh A Oronje R Provision and Use of Maternal Health Services among Urban Poor Women in Kenya: What Do We Know and What Can We Do? J Urban Health 2008 85 428 442 10.1007/s11524-008-9263-1 18389376 PMC2329740

[B53] Schempf AH Branum AM Lukacs SL Schoendorf KC Maternal age and parity-associated risks of preterm birth: differences by race/ethnicity Paediatr Perinat Epidemiol 2007 21 34 43 10.1111/j.1365-3016.2007.00785.x 17239177

[B54] Reflecting on the Trend: Pregnancy After Age 35 http://www.beststart.org/resources/rep_health/pdf/bs_pregnancy_age35.pdf. 10-16-2012

